# Understanding the Subjective Well-Being of 10-and-11-year Old Foster Children in Wales: Relationships and Their Interconnectedness with Places, Possessions and Activities

**DOI:** 10.1007/s12187-026-10381-8

**Published:** 2026-06-10

**Authors:** Colette McAuley, Donald Forrester, Sally Holland, Dawn Mannay, Josie Keam, Jennifer Lyttleton-Smith

**Affiliations:** 1 Children’s Social Care Research and Development Centre (CASCADE), School of Social Sciences, Cardiff University, Cardiff, Wales, UK; 2 Cardiff School of Education and Social Policy, Cardiff Metropolitan University, Cardiff, Wales, UK

**Keywords:** Subjective well-being, Relationships, Children in foster care

## Abstract

More than one per cent of Welsh children are in public care, the majority of whom are aged 5–15 years and living in foster care. Well-being is at the heart of the Social Services and Well-Being (Wales) Act 2014. Under the Act, services are mandated to support the well-being of those they work with, including children who are in out-of-home care. The views of the children on what matters to their well-being are understood to be central to decisions about their care. International advances in researching child well-being has led to an understanding of the central importance of children’s subjective well-being. Subjective well-being is how the person evaluates their life and is based on whatever criteria they use to categorise what is most important in their own lives. When well-being is defined by children, the issues identified can often be very different to those that services, professionals or carers focus upon. Understanding children’s subjective well-being necessitates an exploration of what children feel is important to them in their daily lives. Quantitative and qualitative studies on children’s subjective well-being have increased substantially over the past decade. Most have concentrated on children in the general population, with few studies having focused specifically on children in special circumstances. There has been a gap in Wales in exploring the subjective well-being of children in out-of-home care through a qualitative approach. Such an approach would engage the children in direct face-to-face interviews to more fully explore the children’s own conceptualisation of well-being. This paper describes a qualitative study in Wales, using creative methods to engage younger children. An international Children’s Understandings of Well-Being Protocol, predominantly used with children in the general population, was adapted for use with children living in foster care. Twenty-six children from four Local Authorities in Wales volunteered to take part. They were aged 10–11 years and living in stable foster care and participated in multiple research sessions. The children were asked to map the people, places, possessions and activities which they viewed as important to their well-being. This paper concentrates on the children’s responses and their rationalisations. Relationships were found to be central to the children’s conceptualisation of well-being which aligns with earlier research with schoolchildren of similar age. However, these children were negotiating a much more complex set of family and wider relationships. From the children’s accounts, places, possessions and activities all contribute to the building and maintenance of relationships which are of importance to their well-being. Relational practices and the mechanisms to support and enhance them have been elucidated by the detailed children’s responses. The findings are considered from the point of view of contemporary sociological understandings of the family and childhood. They are also placed in the context of relevant UK and international literature.

## Introduction

### Overview of Paper

Well-being is at the heart of the Social Services and Well-Being (Wales) Act 2014 and should therefore be central to practice and policy for children in out-of-home care in Wales. The Act transforms social care by placing the individual’s voice, control and personal well-being at the centre of care delivery. Under the Act, services are mandated to support the well-being of those they work with, including children who are in out-of-home care. Subjective well-being is what the person involved feels about their life and what is important to them in the here and now. When well-being is defined by children, the issues identified can often be very different to those that services, professionals or carers focus upon. Understanding children’s subjective well-being necessitates an exploration of what children feel is important to them in their daily lives.

International advances in researching child well-being has led to an understanding of the central importance of children’s subjective well-being. Subjective well-being has been defined as ‘an umbrella term for different valuations that people make regarding their lives, the events happening to them, their bodies and minds and the circumstances in which they live’ (Diener, 2006:400). Quantitative and qualitative studies on children’s subjective well-being have increased substantially over the past decade (Ben-Arieh et al., 2017; Fattore et al., 2021).

Whilst there has been interest in the subjective well-being of children in the school population in Wales (Hampton et al., 2019; Hampton & McAuley, 2023), these studies have been based on quantitative surveys with children in the general population. There was a need for qualitative studies to provide greater detail on children’s understandings of their well-being in context and to include children who could be marginalised.

The study reported in this paper is the first qualitative study in Wales which has consulted children living in foster care in person about what matters to their well-being. Given that the perspectives of younger children are less often heard in child welfare research, primary school children were chosen as the focus.

In this paper, we start with some contextual background on children in out-of-home care in Wales. Previous research on child well-being and subjective well-being as well as foster care are then summarised, identifying gaps in the evidence base. Consideration of the issues raised in conducting research with children follows. The rationale, aim and methodology of this study are detailed. Results from the child interviews are presented followed by a discussion of the findings in the context of sociological theory and international research findings.

### Contextual Background

In England and Wales, ‘children looked after’ is the term used for children in out-of-home care and retained in this section as related to the legislation.

#### Children Looked After in Wales

Wales has one of the highest rates of children looked after in the UK and this has been the picture for some time (Elliott, 2019; Forrester et al., 2022). As of 31 st March 2024, there were 7,198 children looked after by Local Authorities in Wales. The majority (61%) were aged 5–15 years and most (68%) were living in foster placements (Welsh Government, 2024a). Children looked after in England and Wales refers to those children who are in care on court orders as well as those accommodated on a voluntary basis.

The vast majority (80%) of Welsh children looked after were the subject of Care Orders, which means that their need for care and protection was decided by the courts and the Local Authority shares parental responsibility for them. Latest figures on reasons for admissions to care indicate that just over half (54%) were due to abuse or neglect (Welsh Government, 2024a). Children in Wales have been found to be more likely to be in care if their parents have learning disabilities, have alcohol or substance misuse, have mental ill health, or are experiencing domestic abuse (Hodges & Bristow, 2019). As in many countries, while children looked after are a heterogenous group, concern has been noted about the higher-than-average mental health problems as well as poorer overall educational achievement and attainment (Welsh Government, 2024b). Hence, considerable focus has understandably been placed on improving outcomes for children in care (Social Care Wales, 2021).

Within this challenging context, the Welsh Government introduced the Social Services and Well-Being (Wales) Act 2014 to refocus social services on user voice, early intervention, multi-agency collaboration, and, most pertinently, supporting the well-being of those accessing social care services above all other priorities (Welsh Government, 2014). At launch, the legislation was promoted as offering ‘transformative change’ to social services, with the voice and rights of those requiring services leading care and support design and delivery (Welsh Government, 2013).

The Social Services and Well-Being (Wales) Act 2014 embraces specifically the concept of well-being of service users in the ‘here and now’ and not just outcomes in the future. Under the Act, services are mandated to regularly consult and support the well-being of those they work with, including children who are looked after.

### Previous Research

#### Research on Child Well-Being and Subjective Well-Being

Child well-being as a concept has been the subject of increasing interest and usage over the past two decades (McAuley & Rose, 2010). As a cultural concept, it represents a shifting set of meanings depending on the context (Erhaut & Whiting, 2008). Normative changes and methodological advances acted as driving forces (Ben-Arieh, 2005). The United Nations Convention on the Rights of the Child (1989) emphasised the right of children to have a voice on matters affecting them. The emergence of a sociology of childhood (James & Prout, 2015) introduced the concept of children as social actors with agency, interacting and actively contributing to their own environments. At the same time, there was widespread acceptance of Bronfenbrenner’s ecological model of child development which emphasised the need to consider all aspects of a child’s development and in the context of family, community and the wider environment (Bronfenbrenner, 1979; Bronfenbrenner & Morris, 2006).

Ben-Arieh (2008) argued that incorporating children’s subjective well-being became both a prerequisite and a consequence of the new field of measuring and monitoring well-being. While concerns were raised initially about the reliability and validity of children’s accounts, it is now well accepted that to develop our understanding of child well-being, we need to ask children directly (Ben-Arieh 2010). Relationships are important to children’s well-being (Goswami, 2011). To understand their social and emotional relationships, the views of children are crucial (Ben-Arieh, 2008).

International research on children’s subjective well-being is a relatively recent but rapidly advancing field (McAuley, 2012). Understandably, it has garnered interest from both quantitative and qualitative researchers. Of particular note is the recent development of two global International Society of Child Indicators (ISCI) research initiatives: the international Children’s Worlds Survey (Ben-Arieh et al., 2017) and the qualitative cross-national Children’s Understanding of Well-Being (CUWB) Project (Fattore et al., 2019). Whilst both initiatives have predominantly concentrated on children in the general population, there is growing interest in the inclusion of children who may be vulnerable as well as children in care or with intellectual disability (Delgado et al., 2020; Munos et al. 2024; Mogensen et al. 2025a).

The first Children’s Worlds (CW) study in Wales in 2018 surveyed 2657 schoolchildren in years 6 and 8 (11 and 13 years on average). Compared with other countries, children in Wales tended to report lower than average levels of subjective well-being (Hampton et al., 2019). This was followed by the Childrens Worlds: COVID-19 Supplement survey in 2021 when 727 schoolchildren in years 6 and 8 from 18 schools participated. Changes in the children’s lives included not being able to attend school, online learning and adjustment to living at home most of the time. These adjustments impacted on their education and relationships with family and school friends. As in other countries, COVID restrictions appeared to exacerbate existing inequalities (Hampton & McAuley, 2023).

The first Children’s Understandings of Well-Being (CUWB) study in the UK took place in North England and included 92 schoolchildren aged 11 years attending four schools in areas with varying levels of deprivation and diversity (McAuley, 2019). The CUWB Protocol for interviews (Fattore et al., 2019) was followed and the methodology adapted to include the use of avatars in film-making with the children (McAuley, 2025). The approach was well received by the children. Relationships were found to be central to the children’s concepts of well-being.

However, the subjective well-being of children in out-of-home care had not received a similar level of attention through qualitative inquiry in the UK. Given the success of the CUWB study described above, this study adopted the same methodological approach with similar age of children. However, rather than engaging a general population it focused on children living in foster care. It was recognised that to engage primary school-age children in care in research interviews would require not only an age-appropriate and child-centred design but a trauma-informed approach (Karmakar and Duggal (2024). Further details are provided in the Research Design section. The following section considers what we know already from foster care research to provide some context for the study.

#### Research on Foster Care

There is a vast literature on foster care, most often emanating from the UK, USA and Australia where it is commonly used to provide care for children unable to live with their birth parents for short or longer periods. Considerable interest has historically focused on foster care outcomes and an attempt to identify the factors which influence success or failure (see Pecora et al., 2006; Sinclair et al., 2005). Often the outcome-based studies have been quantitative and large scale. However, more recent researchers have recognised the value of considering the day-to-day experience of the children and not just the placement outcome. Biehal (2014) emphases the importance of the quality of a child’s experience of long-term foster care, arguing that this should be viewed as an indicator of well-being as important as more objective markers such as placement stability. Pithouse and Rees (2015) engaged with this importance of the everyday and their intensive qualitative study in Wales involving ten foster families predominantly fostering adolescents examined the practices in stable foster care. The role of food, the body and space and place were highlighted.

More recently, children looked after in Wales have been consulted personally on their educational experiences (Mannay et al., 2019; Rees & Munro, 2019) and the culture of care and everyday lives in foster care (Rees, 2019). The largest consultation, the Our Lives Our Care Wales survey, highlighted the importance of relationships, support and opportunities to flourish (Selwyn et al., 2018). There have been no qualitative studies prior to this in Wales which specifically focused on the views of younger foster children looked after on their well-being. The following section reflects on the issues raised when embarking on research which prioritises the voices of children.

#### Research Involving Children

Contemporary childhood studies consider the entire approach to the research process, focusing on how children are involved in the research and the extent to which studies can be considered ‘with’ or ‘by’ the children (Mason & Watson, 2014). There is also close attention paid to power differentials in the adult researcher-child dynamic. In recognition of the positioning of children and their rights as citizens, researchers have generated opportunities for dialogical encounters that draw on creative methods. Creative methods aim to enable children to participate in ways that they find accessible and engaging. These methods are innovative, arts-based approaches that go beyond traditional techniques to explore complex, often sensitive, research data. Participative research methods are collaborative, action-oriented approaches which involve the participants as active partners in the research process rather than just subjects. Participative studies with children might use creative methods including drawing, creating maps and photography to document their lives and surroundings. Researchers have reported children using these methods to be more forthcoming than in standard interviews (Holland et al., 2010; Mannay, 2016; Roerig & Evers, 2019). Some suggest that increased engagement may be due to creative activities offering more time for children to think before responding, which might produce fuller and more considered responses (Angell et al., 2015). Working with visual objects and creative methods can provide a ‘displaced element around which to formulate and advance discussion, acting as a kind of ‘third objects’ around which participants and researchers can focus (Clark & Morriss, 2015). One example would be photovoice, a popular method in qualitative participatory research with young people, involving them taking photographs of images to aid reflection on issues significant to them (Woodgate et al., 2017). In practical terms, the use of creative methods can provide for a more comfortable encounter by reducing eye contact. When vulnerable children are involved, this would be especially important in discussing sensitive topics. Where children find reading and writing difficult, they are often well able to share their thoughts through creative methods (McAuley, 2025). Moreover, these approaches are often experienced by children as engaging and fun (Kara et al., 2021). However, confidentiality and anonymity can be challenging where photographs and art work are involved (Crow & Wiles, 2008). With care-experienced children, issues of past trauma, safeguarding and confidentiality may have particular significance (Mannay, 2020). These approaches to working with children informed the methodological approach in this study as discussed in the following sections.

## The Subjective Well-Being of Younger Children Living in Foster Care Study

### Rationale for the study

This study was designed to increase our understanding of the subjective well-being of young children in foster care. The intention was to both identify what young children view as important to their well-being and gain an understanding of the rationalisations for their decisions.

### Aim of the Study and Research Questions

The aim of the study was to increase our understanding of what younger children living in foster care in Wales view as important to their well-being and their reasoning.

Research Question: How do primary school children living in foster care in Wales understand their wellbeing?

The five research sub-questions addressed in this paper:

1. *Who* do the children view as important to their well-being and why?
2. *What* do the children view as important to their well-being and why?
3. *Where* do the children view as important to their well-being and why?
4. *What activities* do the children view as important to their well-being and why?
5. If they had *three wishes about their well-being*, what would they be?

These sub-questions were aligned with the CUWB Protocol areas suggested for exploration (Fattore et al., 2019), with added emphasis placed on the children’s rationalisations.

### Research Design

The study was a qualitative study seeking to both specifically identify *what* 10 and 11 year old children living in foster care view as important to their well-being and to gain an understanding of their rationalisations as to *why* they are important. Alongside that, the study was seeking to know more generally about any wishes the children might have about their well-being. The design was built upon a protocol using creative methods developed by the ISCI Children’s Understanding of Well-Being Project (Fattore et al. 2015; Fattore et al., 2019). The protocol provides guidance on how to interview children about the people, places and things which are important to their sense of well-being. It was used recently with over one hundred 11- year-old general population schoolchildren in a subjective well-being study in England (McAuley, 2019).

The Protocol design and approach were enhanced and further developed for this study with young children living in foster care in Wales. Based on the results of the earlier CUWB research (McAuley, 2019) along with earlier research with younger foster children (McAuley, 1996), the children were additionally asked to include important activities and wishes about their well-being. The children were asked to create a *Personal Well-Being Map* of the people, places, possessions and activities which they viewed as important to their well-being. They were also asked to complete a *Places and Well-Being Map* to focus more specifically on important places and their significance. Finally they completed a *Three Well-Being Wishes* cartoon exercise. This cartoon was of a genie granting them three wishes with spaces for their responses. Further detail is provided below under data generation.

Additional time and support were provided for the children. An introductory session with the foster carers and children was added to ensure that consent to participate was fully informed and to build a working relationship with the children and their foster carers. Whilst the carers were not present in the research interviews, they were being asked to bring and collect the children from sessions. These sessions also enabled the research team to assess the needs of the children for the research interview sessions. Additional support was provided to the children when creating their maps through increased numbers of staff available. This support was non-directional in regard to content but rather limited to finding resources to make their maps. For example, locating pens, paint, glue, pictures in magazines to add to their visual maps. They also provided support to children with writing and spelling when asked by the children to do so.

The study was designed to be as participatory as possible, enabling children to exercise agency and using engaging creative methods. However, given that it adopted a pre-set protocol, it might be more accurate to describe it as a study which had some participatory aspects where children could have more control over the process in terms of how they responded and the extent to which they were able to then lead the conversations around what they produced in the accompanying interviews (Chicken et al., 2025).

#### Research Ethics

The trauma-informed approach adopted anticipated a history of adverse experiences, being alert to any potential signs of distress in the course of the research engagement and providing a safe space to share the experiences that participants chose to share (Karmaker & Duggai, 2024). Building trusting relationships between children and the researchers at the outset was a key part of the process to ensure that children felt comfortable with sharing their views and this rationality was also centralised. This took into account issues of safeguarding, anonymity and effective care of the participants. All foster carers and children had already received detailed Participant Information letters in advance with this information, with age-appropriate versions for the children. These issues were addressed again specifically with all attendees at the Introductory session. The ethical application for the study, including copies of all participant, carer and Local Authority information, was approved by the authors’ institution in January 2023.

#### Research Context

The research project took place in 2023 and 2024 in four partner local authorities. Two were primarily rural with some small towns and pockets of deprivation - with one in the West and one the East of Wales. One was a post-industrial area of South Wales with high levels of deprivation. The fourth was a town in South Wales, with a mixture of areas of deprivation and comparative affluence and a more ethnically diverse population. In consultation with the Local Authorities, it was agreed that we should draw the sample from foster care only since it is the predominant form of out-of-home care across Wales.

#### Recruitment of Sample

All children in foster care in the care of the four partner local authorities and in Years 5 and 6 (10 and 11 years of age) in primary schools in their areas were deemed eligible. The children’s social workers made the decision as to which children would be approached by them based on the stability of the placements and the best interests of the child. The Introductory Information for Foster Carers, the Child Participant Information and the Child Assent Forms provided by the research team were sent to the families by their social workers. Twenty-six children (14 girls and 12 boys) in total from the four Local Authorities volunteered to participate in the study. The sample was predominantly White Welsh, with three children from Asian, Black African, Caribbean or mixed heritage backgrounds. The lack of ethnic diversity aligns with the population demographics in Wales (Welsh Government 2020). All were the subject of Care Orders and all were in stable foster placements. The research team met with the children on non-school days in child-friendly sites (schools or Flying Start Early Years’ Centres) which were selected by each Local Authority. Four sessions were offered for all children: an introductory session, two research sessions and a follow up session.

#### Data Generation

All of the foster carers and children were invited to attend the introductory session with the research team. Two research sessions followed, and the final session focused on consulting children about resources for dissemination. All children were invited to attend all four sessions, with attendance high apart from the final session which coincided with holiday plans for some families. Since this paper is concentrating on the results of the mapping and wishes exercises in the two research sessions, more detail is provided about the format of these.

In the first research session, each child was asked to create a *Personal Well-Being Map* of the people, places, things and activities that are important to them. They could choose to do this any way they preferred. A large selection of art materials and magazines were made available. When they were finished, the researcher asked the children to share the content of their maps and describe why each element was important. The children were also asked to complete a *Three Well-Being Wishes* cartoon. The children completed their maps at tables with individual support regarding resources by research staff. This took place in a large room with the other children all working on their maps at the same time. However the interviews about the map content and well-being wishes were on an individual basis with their dedicated researcher and carried out to ensure privacy. With their permission in advance, these interviews were recorded. Confidentiality was addressed in the introductory session, with the key message to the children being that they should choose what to share on their maps and in the subsequent interviews. Emphasis was placed in all sessions on the children retaining agency. At the end of the first session the children were reminded that the following session would focus on places and well-being. To facilitate this, we offered each child a disposable camera and asked them to take photographs of places where lived or visited which they viewed as important to their well-being. They had the cameras for at least a week.

In the second research session, the children were asked to create a *Places and Well-Being Map* through drawing, using their own photographs or using pictures from magazines. The children completed their maps in a large room with the other children, individually supported by research staff, but again were interviewed by their dedicated researcher on a one-to-one basis to ensure privacy and confidentiality. In general, the children provided much more information in the interviews as they discussed the map details. The maps appeared to be the vehicle, ‘the third object’ (as described by Clark & Morriss, 2015) which brought about the rich conversations. On average, the children completed their maps in twenty minutes and the subsequent individual interviews lasted a similar amount of time.

The mapping exercises and interviews were child-led, with all researchers trained to use non-directive interviewing. A few children chose not to talk about parts of their drawings or to complete the three wishes exercise. The researchers respected their choices. All sessions involved two senior academic research staff from the team.

### Data Analysis

All interviews were recorded and transcribed, apart from those of a couple of children who chose not to be recorded. With their consent, notes were taken and included in the analyses. All responses were uploaded to NVivo analytic software and standard iterative processes were followed for thematic qualitative analysis (Braun and Clarke 2006). Both the transcriptions and the visual data were considered in the analyses. All three lead authors contributed to the development of the emergent themes through regular discussions based on their cultural, disciplinary, policy and practice expertise.

The research team judged that it was particularly important to analyse the maps with the transcriptions as some children told us in interview that they restricted the information on the map about their birth families as they were unsure with whom the maps would be shared. The fact that they constructed their maps in group situations may also have contributed to their desire for privacy at that stage.

In the reporting of results, care has been taken to ensure children’s identity has been protected and confidentiality maintained. All names used are pseudonyms.

## Findings

Three overarching emergent themes arose from the findings:

(i) *relationships as central to children’s subjective well-being, *
(ii) *the significant of places, possessionsand activitiesto subjective well-being*
(iii) *relationships and their interconnectedness with places, possessions and activities*.

In this section we will explore each theme, adding illustrative material from the children on their choices and reasoning.

### Relationships as central to children’s subjective well-being

#### Foster Families

##### Foster Carers

With regard to foster carers, the children’s reasoning for viewing them as important displayed a high level of consistency. Prominent in their reasoning were words such as ‘nice’, ‘kind’ and ‘helpful’. Several children refined ‘nice’ to ‘nice to me’ which conveyed more about the relationship specifically for them. Feeling that they were loved, supported and encouraged by the carers was frequently mentioned by many children:

*And*,* like*,* I love my (foster) family because*,* like*,* they’ve always supported me. When I find the work hard*,* or*,* like*,* whenever I’m sad*,* or like if…if my mum*,* like*,* bails on me*,* like*,* with a contact*,* like they will try and cheer me up.* (Jenny)

Providing care and opportunities for play and development were frequently mentioned.

*They’re really nice to me*,* they take care of me*,* they make me really nice food*,* like steak…they put the swings up on Monday and I like going up on them…They’re the best foster carers in the whole*,* entire world.* (David)

The sense of being part of a family and being loved featured in several of the responses:

(They are) *basically my mum and dad*… *they are my favourite people. Because they take care of me*,* and they love me.* (Chris)

Enjoying life with the foster family and having fun together featured in several responses:

*I like BBQs in the back garden…we had a BBQ on Sunday which was really good*…(because of) *the food*. (John)

##### Children in the Foster Home

Many children described their foster home as the place where they felt happy and safe. In some instances, it was a welcome refuge after the school day:

*Going home* (makes me happy) *because I get to…see all my dogs*,* play toys and play with my…foster brother…he’s eight.* (Brian)

From the children’s comments, we gained some unique views of how they felt about living with the other children in the foster home. These living situations varied enormously. Some children described living with other foster children along with birth children of the foster carers and their own siblings. Most detailed living with or seeing older children of the foster carers. A small number were fostered on their own with no other children in the household. Where they had birth siblings, whether resident with them or not, all distinguished them as their ‘real’ or ‘real life brothers and sisters. Often their foster siblings were included with birth siblings in their list of people who made them happy. Playing with their siblings, doing different activities together and having fun together appeared to be the key to positive relationships, irrespective of relatedness. Older or adult foster siblings who spent time playing with them were very important to them.

*So*,* people who make me happy is A*,* my foster mum; B and C*,* my real-life brothers; and D*,* E and F*,* my foster brother and sister. And then G*,* which is F’s girlfriend…D*,* E*,*F and G are really nice. And I like them because we do different things…and it’s fun.* (Rachel)

##### Pets in Foster Family Home

The majority of the children referred to pets during discussion of their Personal Well-Being Maps. Pets were generally seen as part of the foster family home and appeared to be particularly important to several of the children. Mostly the children referred to pets in the foster home. Often, they described feeling comfort from contact with their dogs and/or cats in the foster family home, with some being allowed into their private space-their bedroom.

*I enjoy our dog X…because when I feel sad …he just comes to see me and then I feel really happy as well. And she loves me as well*,* because I also feed her food sometimes.* (Ronnie)

*When I came down the stairs this morning*,* she (Y*,* the dog) wagged her tail…she comes and follows me if I go in the garden sometimes*. (Emma)

*I like my dogs as well*,* because they’re very snuggly when I get sad.* (Tara)

#### Birth Families

##### Birth Parents

Birth parents were generally seen as important as they were their birth family, part of their identity and life story.

*Because they’re relatives and they’re*,* like*,* the people who made you…*(Jane).

Several children spoke specifically about their birth mother. These children reported positive feelings towards mum, and felt she was important to them. There was evidence of one child’s awareness of the mother’s poverty and protectiveness towards her.

*It* (contact) *goes good*,* really good…every time we go for something to eat. And my mum asks me if I would like any toys…so she gives me money. But…but I give the money back because she doesn’t have to spend it on me.* (John)

Four children spoke specifically about their birth father and his importance to them. However, none of these children had any recent contact with their fathers, either because the father was no longer alive, he had vulnerabilities preventing contact or because he had moved away to a different country. Yet they identified them as important, simply because they were their birth fathers. Physical resemblance to her deceased father was noted by one child with some degree of pride. Concern was also evident for another father who was addicted to drugs and who was now living in poverty.

*I get… I got everything from my dad. I got the nose*,* I got the eyes*,* I got the eyebrows*,* I got the hair. I got the face* …(Wendy).

*My mum and dad broke up a few years ago when they had an argument*,* and … my actual dad is basically lost. And he’s quite… he’s very poor. He’s like*,* on drugs and stuff.* (Jane)

##### Birth Siblings

Most of the children spoke about the importance of their siblings, including both older and younger siblings. The children felt their siblings were very important, and while a small number reported on arguments and fighting with siblings, they often felt proud of them, and reported enjoying spending time with them. Additionally, there was a sense of strongly developed bonds and gratitude between the children and their siblings.

*I love my brother and I… I know he loves me as well.* (Eleanor)

*So basically she* (her sister) *saved our life. Because if she didn’t*,* I’d probably be dead now. She got us into foster care.* (Jane)

A few children lived with at least one of their siblings in their current foster home. However, many had siblings living in other foster homes and had arranged contact visits. Some siblings had recently been adopted from care.

##### Contact with Birth Family Members

In general, contact with birth family members was deemed important by the majority of the children. Most had contact with members of their birth family whom who wished to see. Contact might be with their birth mothers, birth fathers or siblings or a combination of these. For some, however, the level or the type of contact was a source of concern. Again, a small number of children indicated a lack of contact with birth parents or siblings whom they wished to see or a lack of knowledge about where they were living. Whilst there may have been sound reasons for this, the children did not seem to understand why this was the case. This appeared to be negatively affecting their sense of well-being.

#### Friends

Friends were seen as important by many of the children. This included friends at school and friends living nearby. Activities with their friends were detailed. Some of the children did not mention close friends. One child made a wish to have more friends. Where they had close friends, they indicated the characteristics they appreciated: support when feeling sad, trustworthiness and acceptance.

(X is my best friend) *because he’s always really nice to me*,* and whenever I’m sad he just comes up to me and tries to cheer me up.* (David)

*So*,* my friends are really nice. I have a best friend called Y…I can trust him.* (John)

*My friends*,* they mean*,* like*,* the world to me because*,* like*,* they make me feel*,* like*,* I’m …a part of them…* (Polly).

However, several children appeared to have much more superficial relationships with peers in school and in the neighbourhood and a few expressed anxiety about bullying.

### The Significance of Possessions, Places and Activities to Subjective Well-Being

#### Possessions

Some of the children identified possessions that were important to them. This included electronic devices (e.g. phone, games consoles, iPads, TV), favourite books, flowers, and sensory objects (e.g. slime). Often these were used as a means of keeping in touch with friends. The children also identified objects that provided them with comfort, including blankets, certain items of clothing, family pictures, stuffed toys, and teddy bears. For some children, these possessions held sentimental value related to birth family or early childhood memories.

*I’m going to start on my favourite… my most important*,* which is my… my most important thing which is my blanket I’ve had ever since I was born…and I’ve never washed it because it’s really special and it has like*,* a scent that is like*,* really*,* really like home*,* warming and special to me.* (Alice)

One boy drew pictures of his teddies and explained their importance to him.

*Because when I used to live with my mum and dad*,* they bought me some when I was little.* (Warren)

Another child described the stuffed animals she had in her bedroom, some of which were given to her by her birth mother and sister in the past and the role they play in comforting her.

*I don’t play with them. I just get them down and cover myself with them…(*I feel) *like I have something to hold on to.* (Sheila)

#### Places

The foster home was identified as important by most children as it was a place where they felt loved, cared for and safe. School was viewed by many of the children as important primarily due to contact with their friends there, the support of some teachers and the subjects they were interested in and felt they were good at. Outdoor places and nature were appreciated for walks with their foster families and friends. Places associated with valued contacts visit with birth family were deemed important. Food outlets were popular, with food being highly valued by many of the children. Holidays places featured as important. These were usually with their foster carers and might be abroad (often for the first time), in Wales (in the carers’caravan at well-known beauty spots) or somewhere else in the UK. Two children identified holiday spots associated with their place of birth or with a visit with their birth mother. Further details on the foster home and school follow since they were most frequently identified.

##### Foster Home

The foster home was identified as an important place most frequently by the foster children on both maps. The children described their foster homes as being places of safety, which provided them with a space to play, have positive relationships, access good food, a place to sleep, and a place for their belongings and important possessions. Their bedrooms provided a sanctuary, providing a safe space to relax. For many children, their foster family was what made the home an important place for them, with the children reporting important family activities they do together within the home including family meals, BBQs, parties, and board games.

*So*,* this is my house… I like living there and it makes me happy.* (Eleanor)

*I like being home. I find it my safe place… I really just like it there. I’ve got my room and we all just sit in the living room and talk and eat food together.* (Jenny)

##### School

In both maps, the children talked about how important school was to them. They enjoyed activities within class as well as extra curricula school-based activities. For example, this included having animals in school they could learn about, playing an instrument, singing in the school choir, and attending school trips to the zoo. They named the subjects they enjoyed learning or were best at, including maths, science, literacy, and expressive arts.

School was also associated with the opportunity to see and play with friends, as often they did not live near them.

*And my school*,* … I love school because it… it’s very fun and I like learning new stuff there*. (Tara)

*I li ke being with my friends*,* playing and*,,,* doing maths at school*,* I enjoy that.* (Jenny)

However, not all school peer relationships were seen as positive. One child raised bullying within school and the impact of such behaviour.

*I don’t like it because it can hurt people’s feelings*,* even though the actual bully thinks it’s going to be a joke and (he’ll) have a little laugh…* (Brian).

He explained that such bullying might involve name calling and sending nasty notes.

#### Activities

##### Outdoor and Indoor Activities

When completing their well-being map, the children identified a wide range of activities both inside and outside the home that were important to them.

##### Outdoor Activities

Outdoor activities identified included different forms of sports and exercise, bowling, the cinema, shopping, and church. Children enjoyed forms of exercise, such as walking, cycling, and running, that they could do with the family and surrounded by nature. The children also enjoyed sports which allowed them to master new skills and achieve goals.

*I’ve been horse riding nearly a year. I started at home and then I started to go to horse riding* (classes). *The first thing was holding the horse and then they started teaching you to let go of the horse and then they told me how to control it.*

*It’s because*,* like*,* if we take a walk or we’re just riding the bike in*,* like*,* the outdoors…you’re taking a break from your device and…you have to go on into nature and I…I think …it’s really nice*. (Alice)

Indoor activities included creative activities (e.g., arts and crafts, LEGO*, baking), playing computer games, reading, listening to music, and playing family games. Video games were most frequently reported as an important activity. Gaming was reported to be an activity shared with other people in the home (e.g., siblings), or a way to connect with friends outside school. Arts and crafts was the second most frequently reported activity. For some children, their love for arts and crafts was associated with family members with whom they were currently or had previously engaged in creative activities. For other children, art was important in calming them, giving them a sense of freedom, allowing them to be creative and to express themselves and use their imagination. Dance and playing an instrument were also associated with learning, fun, relaxation or entering another world.

*I like playing with my Switch with my foster brother or my foster sister*,* and I like playing Minecraft with my foster sister.* (David)

*I have made loads of paintings…it’s just relaxing.* (Tara)

#### Well-Being Wishes Concerning Relationships, Possession, Places and Activities

All of the well-being wishes concerned relationships, possessions and activities. Twenty-two children completed the Three Wishes Cartoon. The other four chose not to do so.

Almost one half (10/22) of the children included one or more wishes about their birth families. The wishes were mostly concerns to see or see more of both parents, their mother or their siblings. One child wished for a chance to see her deceased father one more time and another wished for her recently adopted brother to come and live with her again. One child wished she could return to live with her mother.

*Jonathan* (adopted brother) *to come and live with me again.* (Rachel)

*Seeing my sisters more.* (Robert)

*To see my mum and dad once each month.* (David)

*To go back to my mum.* (Charlotte)

Four children had wishes about their foster family. These included that they would live well; that they could live with their foster family forever; and that their foster carers would adopt them. Of particular interest, the child who wished to live with their foster family forever also wished to see birth parents on a regular monthly basis.

*To make sure all the people who look after me live well.* (Ronnie)

*To live with my foster family forever.* (David)

*For Louise and John* (current foster carers*) to adopt me.* (Danny)

Other wishes concerning relationships included one child wishing to see her friends. Two children made more general heartfelt wishes.

(That there were) *no mean people in the world. (Brian)*

*I wish the world had no fights*,* no bad words and no bad people. (Wendy)*

The first child had raised school bullying and its impact whilst the second child had experienced family drug addiction.

Three of the children expressed wishes concerning pets, with two wishing to have a cat and another wishing the dog in his foster home (with whom he had a strong bond) would live a long time.

Wishes for material things and money featured often. Five children wished to be rich whilst other material wishes included to have a mansion, their own house, a pool, unlimited wi-fi, an Xbox and a TV.

Aspirational wishes regarding activities included to be a professional football player; to play football for Liverpool, to be better at dancing; to play rugby for Wales.

### Well-Being Wishes Concerning Relationships, Possession, Places and Activities

From the children’s accounts in this study, it was clear that relationships, places, possessions and activities all appear to contribute to the children’s concept of well-being. Moreover, all of the dimensions also appear to be interconnected. Further, it seemed that relationships appeared to be the core around which the other dimensions revolved.

Activities were often linked to places and people and pets and appeared to be useful for *building new relationships in the foster family*:

*We all just sit in the living room and talk… and eat food together.* (Jenny)

*Going home* (makes me happy), *because I get to …see all my dogs*,* play toys and play with my… foster brother…he’s eight.* (Brian)

*And then me and X* (foster mother) *did a fairy garden. So then in the first part of it we got a butterfly feeding house area. And the fairies - there was one sitting on the swing and one sitting by a sign saying Playground Fairies. And we have a little birdbath there too.* (Rachel)

*She (the dog) comes and follows me if I go in the garden sometimes*. (Emma)

Activities and places were also part of *maintaining relationships* with birth family and friends at school:

*It* (contact with birth mother) *goes good*,* really good…every time we go for something to eat*. (John)

*I like being with my friends playing and… doing maths at school*. (Jenny)

Possessions and relationships were connected too. Phones were linked to *communicating* with friends. Minecraft was a favourite *game which was shared* with other children in the foster home or friends outside school.

*I like playing with my Switch with my foster brother or my foster sister*,* and I like playing Minecraft with my foster sister.* (David)

Possessions could also be things with *significant sentimental value* (for example, photographs, soft toys or a blanket) *related to people and places in their earlier lives*. In the context of separation from their birth families and often multiple placement moves whilst in care, having some possessions to take with you from home to home must have added significance.

*Pictures are important to me because it reminds me of the past… And this is my old doggie- my teddy- which I have had all my life. I have had him since I was two years old.* (Sonya)

However, activities had another dimension: they were valued in themselves for *skill development and the building of competence and confidence*. Learning ‘new stuff’ at school was included here. Also, outside of school, increasing skills in dancing, playing the flute or playing football were seen as important. *Often these skills were linked to their aspirations*, such as to play football for Liverpool or rugby for Wales.

*I really like sport*,* like football and rugby*,* tennis and stuff like that…when I’m older I want to be a goalie*. (David)

Frequently the children described feeling happy whilst completing these activities, with one child describing it was like ‘entering another world’. There was a *sense of sheer joy and of abandonment*, engrossed in these opportunities to flourish.

*Ballet*,* like*,* helps me to relax and it’s just really fun because you’re learning different things.* (Rachel)

*I enjoy playing the flute*,* because it’s like a whole other world really…when I actually play the flute*,* I actually feel happy.* (Ronnie)

## Discussion

Contemporary sociological understanding of the family provides a useful framework to consider the complex experiences of children who have been separated from their primary carers and may have lived in multiple family settings (Holland & Crowley, 2013; Holland et al., 2024). Rather than analysing families as a structure or institution in society, families are understood as constituted through practices which are constructed, relational, active and negotiated (Morgan, 1996, 2011). The children’s accounts in this study include many descriptions of the daily practices in the foster family which they associate with feeling a part of the foster family. For example, walking in the park, having a BBQ in the garden, playing family games at home or simply watching television together. On a more emotional level, foster carers demonstrating support when they are having difficulties at school or when contact arrangements fail are building trustful relationships. Providing daily care, especially ‘nice meals’, was evidence of their commitment to them. Ensuring they had plenty of opportunities for skill development (e.g. arts, drama, music) demonstrated their support for their overall development. Several children indicated that they felt loved by their foster carers and many associated their foster home as the place they felt secure and safe, important issues consistently raised in earlier foster care research (Pithouse & Rees, 2015; Rees, 2019). Most of the children had regular ongoing contact with birth families which was supported by their foster carers. These carers understood the significance of contact for the child and demonstrated this to them. All of which are testament to the family practices in the foster home which helped the children to settle into the family and feel accepted.

Childhood sociologists view children as social actors with agency. In other words, they are active participants in the making and doing of family relationships (James & Prout, 2015). In this study, we can clearly see evidence of the children making decisions about what to share, when and with whom especially about their birth family. Although they may have lived apart from their birth parents and siblings for many years, this was the most emotive area within the child interviews. This was well demonstrated within the Three Well-Being Wishes when over half of the children’s wishes were about birth family. Several children opted out of the exercise and there were indications that it would simply be too painful for them. There was also evidence of decision making about what could be shared about birth family with their foster carers or others. Apart from asserting their right to privacy, it conveyed a level of protectiveness about their birth family. Alongside this there was evidence from one child of a recognition of the vulnerability of their birth parent, particularly with regard to poverty, which made him respond more sensitively to her. In an earlier research study with primary school children in foster care, there was evidence of several children ‘compartmentalising’ details of contact visits and information about each family. This appeared to be both their way of protecting their birth families and personally navigating their relationships with two families in their daily lives (McAuley, 1996).

This study has provided new information from young children in foster care about their experience of living with a myriad of children of different ages. Some were birth siblings who were fostered, some were unrelated children who were fostered and others were birth children of the foster carers. In a few cases, all of these were present. Many had older children of the foster family living in the home or visiting regularly. There was a sense that the study foster children had worked hard to develop good relationships with all of these children and to be accepted through the practices they engaged in. The key to positive relationships appeared to be playing or doing activities together, irrespective of relatedness. The need for more research on how children living in foster care navigate family identities and relationships in foster care has been highlighted (Wulleman et al., 2025).

Pets in the foster home also played a significant role for many of the children who viewed them as part of the family. Mason and Tipper (2008) suggested that children may create their ‘families’ of people and pets through positive interaction (practices). More recent writers emphasise the emotional value of pets to young people in foster care (Pithouse & Rees, 2015). A further qualitative study examined the potential of animals to serve as attachment figures in the context of long-term foster care of children with known attachment difficulties (Carr and Rockett 2017). In our study, there was evidence of many of the children seeing the foster family dog as a safe pal, providing comfort and a sense of unconditional acceptance.

Turning now more specifically to the subject of the relationships between the foster children and their birth parents/family and how the findings of this study contribute to the existing literature. This study has consulted young (10–11 years) children living in foster care about their views about what mattered to their well-being. Without prompting, most confirmed birth family was important to them as part of their identity, referring to them as their ‘real family’. For the majority of the children, contact arrangements were in place with family. However some wished to have more frequent contact or to have the type of contact changed (online to face-to-face). Others wished to have contact with other family members. In all the interviews where the children chose to discuss their birth family, the subject of contact was the most emotive for the children. This was particularly the case in the responses to the three wishes. There was little sense of the children having any agency in this area. Rather decisions appeared to be made without their involvement. This extended beyond actual contact to children’s disappointment about a lack of information being provided about wider family whereabouts or the arrival of new half-siblings. This practice impacted significantly on the children’s sense of agency and well-being. This aligns with international evidence on children having little say in child protection decision-making or participation in the process of care (Toros and Falch-Eriksen 2025; Pohl & Pomey, 2025). Understanding the key relationships in a child’s life and their significance to the child would seem to be crucial for assessing contact arrangements. That involves directly asking the children about who matters to them. Even when there are safeguarding issues which would limit contact, the children need to be given explanations and the opportunity to understand decisions. Birth family relationships need to be considered in the longer term, as many care- experienced adults continue to navigate these often complex relationships into early adulthood (Holland et al., 2024).

Contact between children in out-of-home care and their birth families has been the subject of considerable research over time in the UK and internationally. Studies have predominantly focused on the impact of birth family contact on children’s development and placement outcomes. In a recent review of UK and international research on contact, Iyer et al. (2020) examined 49 studies published between 2000 and 2020 and found that few included children’s views. Overall, a consistent finding from the adult respondents was that children in out-of-home care wanted to see more of their families, be involved in contact decisions and have a choice about the people they saw and when. Two important research gaps were identified: namely, the lack of younger children’s views and children’s perspectives on how contact affected their well-being. This study has found that young children in stable foster care view birth family contact as very important to their well-being, both in terms of reinforcing their sense of identity and reducing anxieties about their birth parents and siblings.

In the study of family relations, few qualitative studies exist on sibling relationships (Holmes et al., 2024) yet they are generally the longest relationship a person has in life. In their literature review on contact, Sen and Broadhurst (2011) highlight the protective role of siblings in maintaining a sense of continuity with birth family and buffering the stress associated with separation. However, a significant proportion are separated when they enter public care or at subsequent placement (Sinclair et al., 2005; Wulczyn & Zimmerman, 2005). This may be due to multiple factors including meeting the needs of the individual child and placement availability. Where they are separated, arrangements for contact need to be considered. However, the review authors found evidence that, where children have been directly consulted, they have reported problems in maintaining or achieving satisfactory levels of contact (Kosonen, 1996; Hegar, 2005).

In this study, the majority of children who chose to discuss birth family contact, indicated they had regular contact with siblings. However, a few children who had strong relationships with siblings voiced concerns about contact arrangements following placement changes or adoption of siblings. Also, a few children indicated that information about significant changes about half-siblings had not been shared with them. There was a strong feeling from the children that they had little or no agency with regard to these matters which clearly had an impact on their well-being.

The child’s sense of satisfaction with contact arrangements appears to be crucial. In their analysis of the data from the Your Life Your Care surveys in 42 English and Welsh local authorities, Selwyn and Lewis (2023) have recently reported that children in their survey (4–18 years and in all types of care) emphasised that the frequency of contact was as important to them, if not more so, than the quality. The individual child’s sense of satisfaction with the contact arrangements was the key factor. The authors raised the important point that social workers need to start by understanding the key relationships in children’s lives. From our limited research mapping and related interviews, the children conveyed a great deal about this. Whilst we are conscious that speaking to a researcher and their social worker may be quite different for children, this type of exercise lends itself to increased understanding between the child and the listener.

Relationships outside the family were also identified as important in this study. Friends featured largely in the accounts of many children. The characteristics of those valued friendships included support, trustworthiness and acceptance. However some of the children did not mention close friends and one child made a wish to have more friends. Several appeared to have much more superficial relationships with peers in school and in the neighbourhood and a few expressed anxiety about bullying.

In their recent international literature review on care experience and friendship, Roesch-Marsh and Emond (2021) found that friendship was important in the lives of those with care experience across countries. Available studies indicated that trust is a key issue for these children whilst making and maintaining friendships, similar to children in the wider population (Blieszner & Roberto, 2003). However, as the authors note, the wider context for care experienced children may be multiple placement moves, often accompanied by school and neighbourhood changes. As a result, friendships may be disrupted and it may be difficult to make or invest in new friendships.

Ridge and Millar (2000) examined friendships of care experienced children and young people from the point of view of social exclusion and its consequences when leaving care. Their interviews with 16 children and young people aged 11–19 years confirmed the importance of friends to talk to in confidence, for support and for protection against bullying. School presented the opportunity to make and sustain relationships outside of the care system, though placement instability could mean school changes and the loss of friends. Hence some may have few or no continuing social networks. On leaving care, reliance on the support of friends was regarded by the young people as crucial. With few social connections, they could find themselves vulnerable when transitioning to independence.

An important finding of the Roesch-Marsh and Emond (2021) review was that children’s friendships were not always recognised as valuable by the adults caring for the children and hence efforts to support the children in this area were not prioritised. They make the argument for a re-framing of friendships as a crucial part of normal childhood for all children, including those with care experience. As such, they argue that friendships should be placed centrally in assessment and intervention work. Specific support for the development of friendship practices for children in care was recommended.

Relational practices and the mechanisms to support and enhance them have been elucidated in this study. From the children’s accounts, activities, places and possessions all contribute to the building and maintenance of relationships which are of importance to their well-being.

Family activities such as having meals together, going on walks, playing games together all contributed to building new relationships in the foster home, not just with the foster carers but also with the range of children living there. Shared activities can provide the context for children to learn and negotiate relationships (McAuley et al., 2012). Again, activities and places were associated with maintaining relationships with birth families and siblings on contact visits. Playing together with friends especially at school helped to build and maintain relationships. Outdoor places and nature were appreciated and there is evidence that time spent in nature generally enhances well-being and has positive effects (Adams & Savahl, 2017; Arola et al., 2023).

Possessions such as phones and games were often used for communication and sharing with other children in the foster home and friends. Those photos, blankets and toys with sentimental value were keeping alive memories of people and places in their earlier lives. Ward and colleagues (2006) have already drawn attention to the significance of such possessions for children in out-of-home care, having been separated from birth family and often experiencing multiple moves subsequently whilst in care.

Overall, the children’s responses suggested the interconnectedness of relationships, activities places and possessions. However, relationships appeared to be of central importance to their subjective well-being.

### Concluding Comments

In any exploration of well-being, it is important to consider the factors which lead people to think and experience their lives in positive or negative ways-the attributes of subjective well-being (Diener et al., 2018). Accordingly, subjective well-being is based on whatever criteria individuals use to categorise what is most important in their own lives. When well-being is defined by children, the issues identified can often be very different to those that services, professionals or carers focus upon and their rationalisations often little known. This study has centralised the importance of the subjective well-being of the foster children.

From a sociological point of view, the children’s accounts in this study have reinforced the view of children as social actors with agency contributing to the relational practices within their foster families and beyond. However, their responses have to be viewed as situated within the contexts in which they live and may or may not be similar to those of younger children looked after in different contexts. The challenges of cross-national comparison of qualitative studies have been the subject of recent debates (see Fattore et al. 2021; Mogensen et al. 2025b).

With regard to policy and practice, well-being has been placed at the heart of the Social Services and Well-Being (Wales) Act 2014. One way to operationalise this in practice might be the introduction of well-being conversations into the statutory six-monthly review process for each child in out-of-home care. The use of creative methods, such as well-being maps, with younger children as part of review preparation might well enhance their level of participation in reviews where important decisions are made about their lives.

### Limitations

The Local Authorities selected the children for participation in the study. The decisions were based on the children’s interest in participating and the support of their foster carers and social workers. The children were in stable placements. Hence the study does not seek to be representative of the views of children living in foster care more generally. Rather the accounts need to be considered in this context.

## Data Availability

The sensitive nature of the qualitative data and the agreement with the participants prevent the sharing of participants’ information. To protect participant confidentiality, specific details will not be disclosed or shared publicly.

## References

[CR1] Adams, S., & Savahl, S. (2017). Nature as children’s space: A systematic review. *The Journal of Environmental Education,**48*(5), 291–321.

[CR2] Angell, C., Alexander, J., & Hunt, J. A. (2015). Draw, write and tell: A literature review and methodological development on the ‘draw and write’ research method. *Journal of Early Childhood Research*, *13*, 17–28.

[CR3] Arola, T., Aulake, M., Ott, A., Lindholm, M., Kouvonen, P., Virtanen, P., & Paloniemi, R. (2023). The impacts of nature connectedness on children’s well-being: Statematic literature review. *Journal of Environmental Psychology,**85*, Article 102913.

[CR5] Ben-Arieh, A. (2005). Where are the children? Children’s role in measuring and monitoring their well-being. *Social Indicators Research,**74*(3), 573–596.

[CR6] Ben-Arieh, A. (2008). The child indicators movement: Past, present and future. *Child Indicators Research*, *1*, 3–16.

[CR4] Ben Arieh, A. (2010). Developing indicators for child well-being in a changing context. In C. McAuley, & W. Rose (Eds.), *Child well-being: Understanding children’s lives* (1st ed., pp. 129–142). Jessica Kingsley.

[CR7] Ben-Arieh, A., Dinisman, T., & Rees, G. (2017). A comparative view of children’s subjective well-being: Findings from the second wave of the ISCIWeb Project. *Children and Youth Services Review*, *80*, 1–2.

[CR8] Biehal, N. (2014). A sense of belonging: Meanings of family and home in long-term foster care. *British Journal of Social Work*, *44*, 955–971.

[CR9] Blieszner, R., & Roberto, K. A. (2003). Friendship across the lifespan: Reciprocity in individual and relationship development. In F. R. Lang & K. Fingerman (Eds.), *) Growing together: Personal relationships across the lifespan* (pp. 159–82). Cambridge University Press.

[CR10] Braun, V., & Clark, V. (2006). *Thematic analysis: A practical guide*. Sage.

[CR11] Bronfenbrenner, U. (1979). *The ecology of human development*. Cambridge University Press.

[CR12] Bronfenbrenner, U., & Morris, P. (2006). The bio-ecological model of human development. In R. M. Lerner, & W. Damon (Eds.), *Handbook of child psychology: Theoretical models of human development* (6th ed., pp. 793–828). John Wiley &Sons.

[CR13] Carr, S., & Rocket, B. (2017). Fostering secure attachment: Experiences of animal companions in the foster home. *Attachment and Human Development*, *19*(3), 259–277. 28277096 10.1080/14616734.2017.1280517

[CR14] Chicken, S., Tur Porres, G., Mannay, D., Parnell, J., & Tyrie, J. (2025). Questioning ‘voice’ and silence: Exploring creative and participatory approaches to researching with children through a Reggio Emilian lens. *Qualitative Research,**25*(1), 3–20.

[CR15] Clark, A., & Morriss, L. (2015). The use of visual methodologies in social work research over the last decade: A narrative review and some questions for the future. *Qualitative Social Work,**16*(1), 29–43.

[CR16] Crow, G., & Wiles, R. (2008). *Managing anonymity and confidentiality in social research: The case of visual data in community research*. ESRC National Centre for Research Methods NCRM Paper Series 8/08.

[CR17] Delgado, P., Carvalho, J. M. S., Montserrat, C., & Llosada-Gistau, J. (2020). The subjective well-being of Portuguese children in foster care, residential care and children living with their families: Challenges and implications for a child care system still focused on institutionalisation. *Child Indicators Research*, *13*, 1.

[CR18] Diener, E. (2006). Guidelines for national indicators of subjective well-being and ill-being. *Journal of Happiness Studies*, *7*, 397–404.

[CR19] Diener, E., Oisi, S., & Tay, L. (2018). Advances in subjective well-being research. *Nature Human Behaviour*, *2*, 253–260. 10.1038/s41562-018-0307-6 30936533

[CR20] Elliott, M. (2019). Charting the rise of children and young people looked after in Wales. In D. Mannay, A. Rees, & L. Roberts (Eds.), *Children and young people ‘looked after’? Education, intervention and the everyday culture of care in Wales* (1st ed., pp. 15–28). University of Wales Press.

[CR21] Ereaut, G., & Whiting, R. (2008). *What do we mean by ‘wellbeing’? And why might it matter? Research Report DCFS-RW073*. Department for Children, Schools and Families.

[CR22] Fattore, T., Fegter, S., & Hunner-Kreisel, C. (2019). Children’s understandings of well-being in global and local contexts: Theoretical and methodological considerations for a multinational qualitative study. *Child Indicators Research*, *12*(5), 385–407.

[CR23] Fattore, T., Fegter, S., & Hunner-Kreisel, C. (2021). *Children’s concepts of well-being: Challenges in international comparative qualitative research.* Springer Nature.

[CR63] Forrester, D., Wood, S., Waits, C., Jones, R., Bristow, D., & Taylor-Collins, E. (2022). *Children’s social services and care rates in Wales: A survey of the sector*. Wales: Centre for Social Policy.

[CR24] Goswami, H. (2011). Social relationships and children’s subjective well-being. *Social Indicators Research*, *3*, 575–588.

[CR25] Hampton, J., & McAuley, C. (2023). The impact of COVID-19 on well-being: Welsh children’s perspectives. *International Journal on Child Maltreatment,**6*, 477–488. 10.1007/s42448-023-00149-w PMC10026787 37360285

[CR26] Hampton, J. M., Power, S., & Taylor, C. (2019). *Children’s worlds national report Wales.* Welsh Institute of Social and Economic Research.

[CR27] Hegar, R. (2005). Sibling placement in foster care and adoption: An overview of international research. *Children and Youth Services Review*, *27*, 845–861.

[CR28] Hodges, H., & Bristow, D. (2019). *Analysis of the factors contributing to the high rates of care in Wales*. Wales Centre for Public Policy.

[CR64] Holland, S., & Crowley, A. (2013) Looked after children and their birth families: Using sociology to explore changing relationships, hidden histories and nomadic childhoods. *Child and Family Social Work, 18,* 57–66.

[CR65] Holland, S., Renold, E., Ross, N., & Hillman, A. (2010). Power, agency and participatory agendas: A critical exploration of young people’s engagement in participative qualitative research. *Childhood, 17(*3), 360–375.

[CR66] Holland, S., Taussig, H., & Havlicek, J. (2024). Hurt, loss, joy and forgiveness: Foster care-experienced young adults’ relationships with their birth parents. *Family Relations, 74*(4), 2000–2016. 10.1111/fare.13184 PMC12383907 40881135

[CR29] Holmes, M. R., Bender, A. E., O’Donnell, K. A., Miller, E. K., & Conard, I. T. (2024). Illuminating the landscape of sibling relationship quality: An evidence and gap map. *Child Development,**95*(4), 1425–14440. 38185938 10.1111/cdev.14065

[CR30] Iyer, P., Boddy, J., Hammelsbeck, R., & Lynch-Huggins, S. (2020). *Contact following placement in care, adoption or special guardianship: Implications for children and young people’s well-being. Evidence review.* Nuffield Family Observatory.

[CR31] James, A., & Prout, A. (2015). *Constructing and reconstructing childhood: Contemporary issues in the sociological study of childhood.* (3rd ed.). Routledge.

[CR32] Kara, H., Lemon, N., Mannay, D., & McPherson, M. (2021). *Creative research methods in education: Principles and practices.* Policy Press.

[CR33] Karmakar, S., & Duggal, C. (2024). Trauma-informed approach to qualitative interviewing in non-suicidal self-injury research. *Qualitative Health Research*, *34*(1–2), 33–47. 37924212 10.1177/10497323231207746

[CR34] Kosonen, M. (1996). Maintaining sibling relationships-neglected dimension in child care practice. *British Journal of Social Work*, *26*, 809–822.

[CR67] McAuley, C. (1996). Children’s perspectives on long-term foster care. In M. Hill & J. Aldgate (Eds.), *Child welfare services: Developments in law, policy, practice and research* (1st ed., pp. 170–179). Jessica Kingsley.

[CR68] McAuley, C. (2012) Editorial. International advances in child well-being: Measuring and monitoring subjective well-being. *Child Indicators Research, 5,* 419–421.

[CR69] McAuley, C. (2019). Exploring eleven year old children’s understanding of well-being using well-being maps: Commonalities and divergences across areas of varying levels of deprivation and ethnic diversity in an English Qualitative Study. *Children and Youth Services Review, 97,* 22–29.

[CR70] McAuley, C. (2025). Well-being maps and the introduction of avatars in filmmaking: Reflections on ethics and visual methods in an English qualitative study of child well-being. In L. Mogensen, S. Fegter, S. L. Fischer, J. Mason & T. Fattore (2025) *Qualitative fieldwork with children: Context and participation in child well-being research across nations* (1st ed., pp. 244–263). Bristol University Press.

[CR77] McAuley, C. and Rose, W. (2010). *Child well-being; Understanding children’s lives*. London: Jessica Kingsley.

[CR78] McAuley, C., McKeown, C., & Merriman, B. (2012). Spending time with family and friends; Children’s views on relationships and shared activities. *Child Indicators Research*, *5*, 449–467.

[CR73] Mannay, D. (2016). *Visual, narrative and creative research methods: Application, reflection and ethics*. London: Routledge.

[CR74] Mannay, D. (2020). Revisualizing data: Engagement, impact and multimodal dissemination. In L. Pauwels & D. Mannay (Eds.), *The sage handbook of visual research methods* (2nd ed., pp. 659–669). Sage.

[CR75] Mannay, D., Rees, A., & Roberts, L. (Eds.) (2019) *Children and young people ‘looked after’? education, intervention and the everyday culture of care in Wales*. Cardiff: University of Wales Press.

[CR35] Mason, J., & Tipper, B. (2008). Being related: How children define and create kinship. *Childhood,**15*(4), 441–460.

[CR36] Mason, J., & Watson, E. (2014). Researching children: Research on, with and by children. In A. Ben-Arieh, F. Casas, I. Frones, & J. Korbin (Eds.), *Handbook of child well-being: Theories, methods and policies in global perspective* (Vol. 5, pp. 2757–2796). Springer.

[CR37] Mogensen, L., Drake, G., Michail, S., Fattore, T., Falloon, J., & Mason, J. (2025a). Navigating child well-being research in institutional settings: Children with intellectual disability and children in care. In L. Mogensen, S. Fegter, L. Fischer, J. Mason, & T. Fattore (Eds.), *Qualitative fieldwork with children: Context and participation in child well-being research across nations* (1st edition, pp. 127–147). Bristol University Press.

[CR38] Mogensen, L., Fegter, S., Fischer, L., Mason, J., & Fattore, T. (2025). *Qualitative fieldwork with children: Context and participation in child well-being research across nations.* Bristol University Press.

[CR39] Morgan, D. (1996). *Family connections: An introduction to family studies*. Polity Press.

[CR40] Morgan, D. (2011). *Rethinking family practices*. Palgrave Macmillan.

[CR41] Munoz, M. S., Montserrat, C., Alfaro, & Melipillan, R. (2024). Subjective well-being of vulnerable children in Chile: Differences by gender and risk assessment. *Child Indicators Research*, *18*, 115–135.

[CR42] Pecora, P., Kessler, R. C., O’Brien, K., White, C. R., Williams, J., Hiripi, E., English, D., White, J., & Herrick, M. A. (2006). Educational and employment outcomes of adults formerly placed in foster care: Results from the Northwest foster care alumni Study. *Children and Youth Services Review,**28*(12), 1459–1481.

[CR43] Pithouse, A., & Rees, A. (2015). *Creating stable foster placements: Learning from foster children and the families who care for them*. Jessica Kingsley Publishers.

[CR44] Pohl, C., & Pomey, M. (2025). The interconnectedness of vulnerability and agency from the perspective of children in child and youth services. *European Journal of Social Work*, *27*, 798–809.

[CR45] Rees, A. (2019). The daily lived experiences of foster care: The centrality of food and touch in family life. In D. Mannay, A. Rees, & L. Roberts (Eds.), *Children and young people ‘looked after’? Education, intervention and the everyday culture of care in Wales* (1st ed., pp. 85–98). University of Wales Press.

[CR76] Rees, P., & Munro, A. (2019). Promoting the education of children in care: Reflections of children and carers who have experienced ‘success’. In: D. Mannay, A. Rees, & L. Roberts (Eds), *Children and young people ‘looked after’? Education, intervention and the everyday culture of care in Wales* (1st ed., pp. 56–68). University of Wales Press.

[CR46] Ridge, T., & Millar, J. (2000). Excluding children; autonomy, friendship and the experience of the care system. *Social Policy & Administration,**34*(2), 160–175.

[CR47] Roerig, S., & Evers, S. (2019). Theatre elicitation: Developing a potentially child-friendly method with children aged 8–12. *Children’s Geographies,**17*, 133–147.

[CR48] Roesch-Marsh, A., & Emond, R. (2021). Care experience and friendship: Theory and international evidence to improve practice and future research. *British Journal of Social Work*, *51*, 132–149.

[CR49] Selwyn, J., & Lewis, S. (2023). Keeping in touch: Looked after children and young people’s views on their contact arrangements. *Adoption & Fostering,**47*(2), 120–137.

[CR50] Selwyn, J., Magnus, L., Symonds, J., & Briheim-Crookal, L. (2018). *Our lives Our care Wales 2018.* University of Bristol and Coram Voice.

[CR51] Sen, R., & Broadhurst, K. (2011). Contact between children in out-of-home placements and their family and friends networks: A research review. *Child & Family Social Work,**16*, 298–309.

[CR52] Sinclair, I., Wilson, K., & Gibbs, I. (2005). *Foster placements: Why they succeed and why they fail*. Jessica Kingsley Publishers.

[CR53] Social Care Wales. (2021). *Improving outcomes for children programme-Legacy report.* Welsh Government.

[CR54] Toros, K., & Falch-Erikson, A. (2025). “I got to say two or three lines”-a systematic review of children’s participation in child protection services. *Child Abuse & Neglect,**162*, Article 106934. 38971702 10.1016/j.chiabu.2024.106934

[CR55] Ward, H., Munro, E., & Dearden, C. (2006). *Babies and young children in care: Life pathways, decision-making and practice.* Jessica Kingsley.

[CR58] Welsh Government. (2013). *Social Services and Well-Being (Wales) Bill, Explanatory memorandum*. Welsh Government.

[CR59] Welsh Government. (2014). *Social Services and Well-being (Wales) Act 2014*. Welsh Government.

[CR56] Welsh Government (2024a). *Children looked after by local authorities: April 2023 to March 2024*. Available at https://www.gov.wales/children-looked-after-local-authorities-april-2023-march-2024*.* [ Accessed 18 February 2025].

[CR57] Welsh Government (2024b). *Children receiving care and support census: On 31 March 2024*. Available at https://www.gov.wales/children-receiving-care-and-support-census-31-march-2024. [Accessed 18 February 2025].

[CR60] Woodgate, R. L., Zurba, M., & Tenent, P. (2017). Worth a thousand words? Advantages, challenges and opportunities in working with photovoice as a qualitative research method with youth and their families. *Forum: Qualitative Social Research*, 18.

[CR61] Wulczyn, F., & Zimmerman, E. (2005). Sibling placements in longitudinal perspective. *Children and Youth Services Review,**27*(7), 741–763.

[CR62] Wulleman, L., Grietens, H., Noens, I., & Vliegen, N. (2025). Roots and routes: Navigating family identities and relationships in non-kinship care. *Children and Youth Services Review,**172*, Article 108290.

